# 1112. Ceftobiprole for the Treatment of Invasive *Enterococcus faecalis* Infections: Ampicillin, "Should I Stay or Should I Go?"

**DOI:** 10.1093/ofid/ofad500.085

**Published:** 2023-11-27

**Authors:** Simone Giuliano, Denise D’Elia, Jacopo Angelini, Alberto Pagotto, Sarah Flammini, Floriana Campanile, Carlo tascini

**Affiliations:** Infectious Diseases Division, Department of Medicine, University of Udine and Azienda Sanitaria Universitaria Friuli Centrale, Udine, Italy, Udine, Friuli-Venezia Giulia, Italy; Infectious Diseases Division, Department of Medicine, University of Udine and Azienda Sanitaria Universitaria Friuli Centrale, Udine, Italy., Udine, Friuli-Venezia Giulia, Italy; Clinical Pharmacology and Toxicology Institute, University Hospital Friuli Centrale ASU FC, 33100 Udine, Italy, Udine, Friuli-Venezia Giulia, Italy; Infectious Diseases Division, Department of Medicine, University of Udine and Azienda Sanitaria Universitaria Friuli Centrale, Udine, Italy, Udine, Friuli-Venezia Giulia, Italy; Infectious Diseases Division, Department of Medicine, University of Udine and Azienda Sanitaria Universitaria Friuli Centrale, Udine, Italy., Udine, Friuli-Venezia Giulia, Italy; Department of Biomedical and Biotechnological Sciences, Section of Microbiology, University of Catania, I-95123 Catania, Italy., Catania, Sicilia, Italy; Infectious Diseases Division, Department of Medicine, University of Udine and Azienda Sanitaria Universitaria Friuli Centrale, Udine, Italy., Udine, Friuli-Venezia Giulia, Italy

## Abstract

**Background:**

*Enterococcus faecalis* is responsible for many serious infections for which combination bactericidal therapy is required^1^. A synergism between amoxicillin and cefotaxime against *E. faecalis* through partial saturation of penicillin-binding proteins (PBPs) 4 and 5 by amoxicillin and total saturation of PBPs 2 and 3 by cefotaxime has been demonstrated^2^. Unlike cefotaxime, ceftobiprole binds with high affinity to PBP2, PBP3, PBP4 and PBP5^3,4,5^, thus a potential role in the management of severe *E. faecalis* infections might be hypothesized. The availability of therapeutic drug monitoring (TDM) for ampicillin and ceftobiprole has been driving the choice of using the combination of ampicillin plus ceftobiprole (ABPR) for the treatment of life-threatening *E. faecalis* infections.

**Methods:**

We retrospectively analyzed clinical features (Table1), and trough ceftobiprole and ampicillin plasmatic concentrations of 21 patients admitted to our hospital from January to December 2020 for serious infection due to *E. faecalis* (61% infective endocarditis and 39% complicated bacteremia) who underwent ABPR treatment. Bacterial killing kinetics were also investigated in vitro for all strains. Bactericidal activity was defined as a ≥3 log10 decrease in bacterial count at 24 h. Synergy was measured at 24 h and was defined as a  ≥ 2 log10 decrease in CFU/mL by the combination compared with its most active constituent and a  ≥ 2 log10 decrease in the CFU/mL below the starting inocula.

**Table 1.**
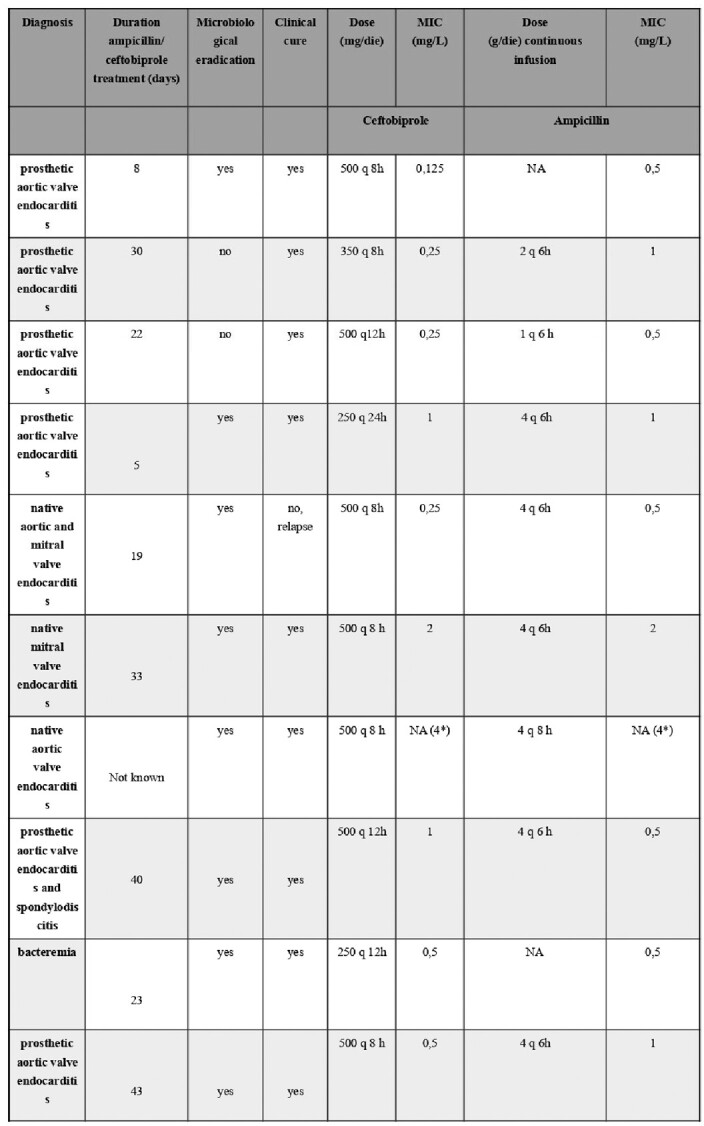
Patient level pathological conditions, clinical and microbiological outcomes and antimicrobial therapy. MIC= minimum inhibitory concentration.

**Figure:**
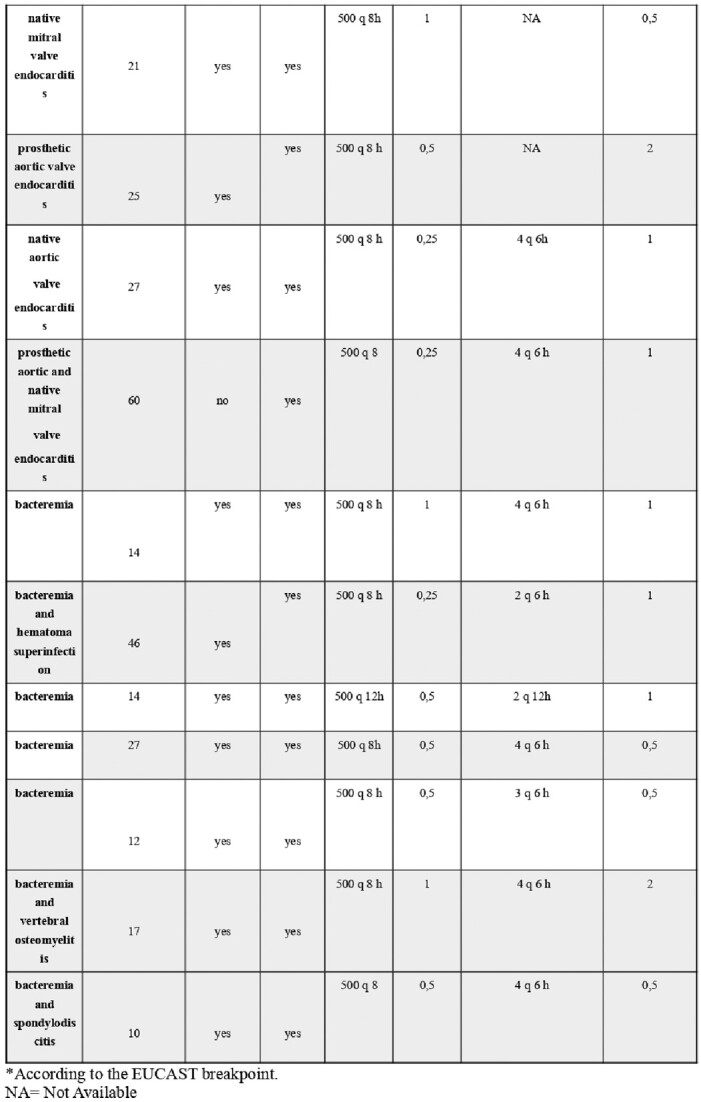

**Results:**

High clinical success rate of 81% and elevated microbiological eradication rate of 86% were reported. The ratios among all patients total plasmatic ampicillin and ceftobiprole concentrations and the corresponding MIC of enterococcal isolates far exceeded the PK/PD targets of maximized bactericidal activity ^6^ ( >4-5) (Figure 1). Ceftobiprole MIC_50_ and MIC_90_ were 0.5 mg/l and 1 mg/l, respectively. Time-kill curve for one of the 21 studied *E. faecalis* isolates is reported in Figure 2. Ceftobiprole showed such a potent bactericidal activity at concentration four times the MIC that a synergistic effect with ampicillin was even not demonstrable (Figure 2).

**Figure 1.**
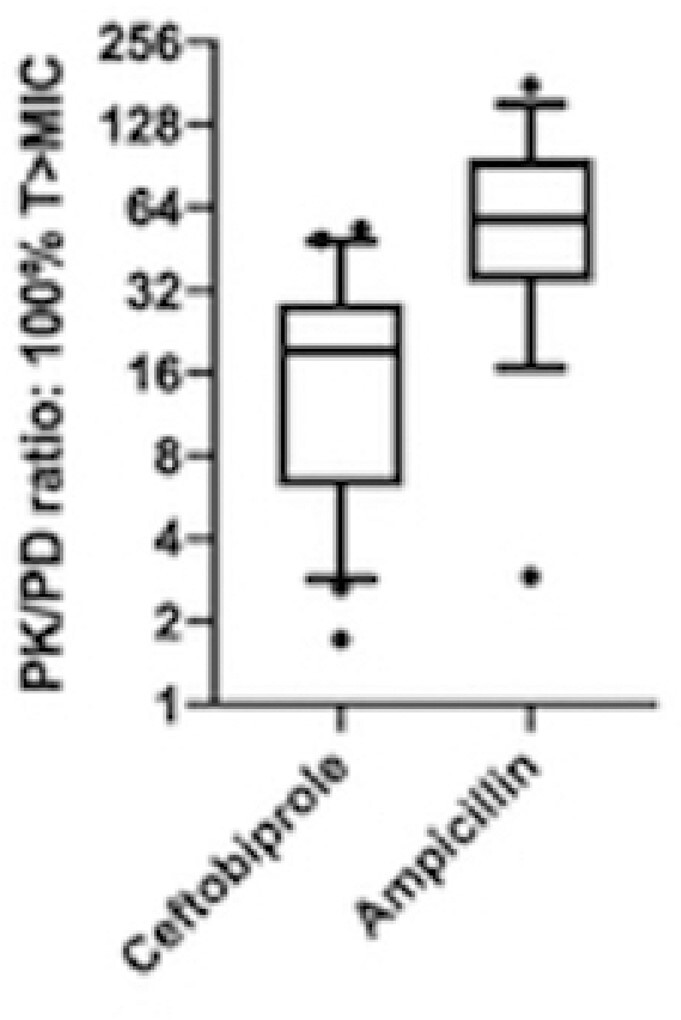
The observed Pharmacokinetic/Pharmacodynamic (PK/PD) antimicrobial efficacy index, which is represented by the ratio between the antibiotic plasmatic concentrations at the end of dosing interval (100% T) and the reference MIC (100%T>MIC). A ratio of 1 represents the minimum PK/PD therapeutic target, whereas the ratio of 4 represents a high antimicrobial activity. The reported MICs derive from pathogen isolates. MIC= minimum inhibitory concentration.

**Figure 2.**
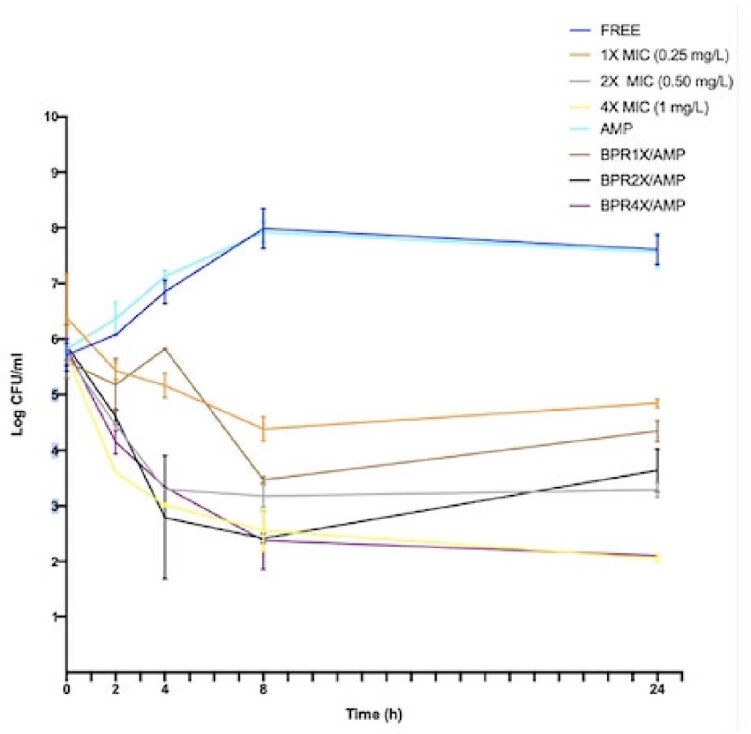
Time–kill curve of ampicillin and ceftobiprole alone and in combination at different BPR concentrations (1 x MIC, 2 x MIC, 4 x MIC) for one of the 21 studied E. faecalis strains. AMP, ampicillin, BPR, ceftobiprole. MIC AMP: 1 mg/L; MIC BPR: 0.25 mg/L.

**Conclusion:**

Ceftobiprole even alone or ABPR might represent a valuable option for the treatment of severe *E. faecalis* invasive infections.

**Disclosures:**

**All Authors**: No reported disclosures

